# Surgical treatment of adolescent internal condylar resorption (AICR) with articular disc repositioning and orthognathic surgery in the growing patient—a pilot study

**DOI:** 10.1186/s40510-015-0115-8

**Published:** 2016-01-18

**Authors:** Trevor P. Bodine, Larry M. Wolford, Eustaquio Araujo, Donald R. Oliver, Peter H. Buschang

**Affiliations:** Department of Orthodontics, Texas A&M University Baylor College of Dentistry, 3302 Gaston Ave, Dallas, TX 75246 USA

## Abstract

**Background:**

The aim of this study was to better understand how surgical repositioning and stabilization of anteriorly displaced articular discs using the Mitek mini-anchor technique affects condylar growth in growing patients with adolescent internal condylar resorption (AICR).

**Methods:**

Twenty-two adolescent patients diagnosed with AICR and anterior temporomandibular disc displacement were compared to untreated control subjects without AICR matched for age, sex, and Angle classification. Pre-surgical (T1 and T2) and post-surgical (T3 and T4) mandibular tracings were superimposed on natural stable structures to evaluate the horizontal, vertical, and total changes in the position of condylion.

**Results:**

The treated group showed an overall decrease in condylar height pre-surgically and statistically significant changes in condylar growth direction between the pre- and post-surgical observation periods. Pre-surgically, the treated group showed significantly more posterior condylar growth than the control group; they also showed inferior condylar growth, while the controls showed superior growth. Controls and patients in the treated group showed no significant differences in condylar growth post-surgically.

**Conclusions:**

Adolescent patients diagnosed with AICR and anterior disc displacement treated with mandibular ramus and maxillary osteotomies, along with Mitek anchors to reposition internally deranged discs, showed post-surgical normalization of condylar growth.

## Background

Temporomandibular joint dysfunction (TMD) is often a complaint of orthodontic patients. TMD problems can manifest themselves in many forms, from simple clicking noises on opening to debilitating headaches, as well as joint pain and dysfunction. Anterior displacement of the articular disc is the most commonly occurring joint dysfunction, affecting nearly 40 % of the general population [1]. Displacement of the disc can occur at any time of life; it commonly follows traumatic events that alter the condyle-disc relationship. Adolescent patients are susceptible to this derangement, with or without a traumatic insult, especially during their rapidly changing growth phases [2–5].

While many treatment modalities have been devised to alleviate the symptoms of temporomandibular joint (TMJ) dysfunction [6–9], few therapies actually correct the pathological process. One treatment modality for the correction of an anteriorly displaced disc utilizes the Mitek mini-anchor technique [10–12]. The mini-anchor is tethered to the posterior band of the disc and embedded in the posterior head of the condyle. The Mitek technique has been shown to be stable and to eliminate TMD symptoms in both adolescents and adults [12–14]. However, TMJ surgery remains controversial when surgically exposing the joint of adolescents. Concerns exist due to the possible effects of surgical manipulation on condylar growth.

The Mitek mini-anchor technique is applicable to cases of disc displacement of various etiologies, including adolescent internal condylar resorption (AICR) [15, 16]. In terms of etiology and clinical presentation, AICR is a distinct TMJ pathology that differs from other conditions causing condylar resorption. AICR causes condylar resorption in all three planes of space by loss of condylar subcortical and cancellous bone, while maintaining the fibrocartilage on the condylar head and in the fossa. There are no other joints involved in AICR and no known genetic predisposition [10, 17]. Onset usually occurs between the ages 11 and 15 years. It predominately affects teenage females during pubertal growth.

The aim of this current study was to assess pre- and post-surgical growth of the condyle in patients with AICR treated with the Mitek mini-anchor system. The study evaluates both the pre-surgical and post-surgical changes that occur to determine whether the condylar growth of the condyle can be altered.

## Methods

### Patient sample

This retrospective study pertains to 22 female adolescent patients diagnosed with AICR who had undergone bilateral temporomandibular articular disc repositioning surgery using the Mitek mini-anchor technique to stabilize and retain the disc position. Concomitant maxillary and mandibular osteotomies for counter-clockwise rotation advancement of the maxillo-mandibular complex were performed by the same surgeon (LMW). All patients were undergoing orthodontic treatment by different orthodontists at the time of surgery. The study was approved by the Institutional Review Board at Saint Louis University.

Patients were chosen based on having the following: (1) growth potential (10 to 16 years of age pre-surgically), (2) a diagnosis of AICR with anterior TMJ disc displacement, (3) no Angle class III malocclusions, (4) no craniofacial anomalies, (5) no previous TMJ or orthognathic surgical procedures, (6) absence of any pre- or post-surgical trauma, and (7) post-surgical records at least 1 year post-op.

The patients had lateral cephalometric radiographs available at four time points (Table 1): initial exam (T1), immediate pre-surgical (T2), immediate post-surgical (T3), and 1-year follow-up exam (T4). The final treatment group consisted of 18 patients with pre-surgical (T1 and T2) records, 15 with post-surgical records (T3 and T4), and 11 patients with full records available (T1, T2, T3, and T4).

**Table 1:** Age descriptive statistics for treatment and control subjects

	Treatment group		Control group	
	Mean (years)	SD	Mean (years)	SD
T1 age	14.59	1.83	14.16	1.83
T2 age	15.16	1.45	15.16	1.83
T3 age	14.97	1.83	14.80	1.69
T4 age	15.93	2.12	15.80	1.69

All patients were diagnosed as having AICR by the same surgeon (LMW). AICR diagnosis was based upon patient history, clinical evaluation, radiographic assessment (lateral cephalogram and TMJ tomograms), MRI, and direct observation at surgery. Patients generally reported a progressive worsening of their occlusion, posterior shifting of their mandible, and development of an anterior open bite, with or without TMJ symptoms or pain. MRI findings showed (1) decreased condylar head volume, (2) anterior disc displacement with or without reduction on opening, and (3) thinning or loss of continuity of cortical bone on the head of the condyle. Cephalometric evaluations showed (1) skeletal and occlusal class II deformities, (2) anterior open bites, (3) high occlusal and mandibular plane angles, and (4) decreased vertical heights of the ramus and posterior maxilla. There are currently no laboratory tests specific for AICR.

All patients in the treatment group began routine orthodontic therapy after the initial exam (T1) and were undergoing orthodontic treatment at the time of surgery. In addition to the Mitek anchor surgery, all patients underwent concomitant maxillary osteotomies and bilateral mandibular ramus sagittal split osteotomies (BSSO) for counter-clockwise rotation advancement of the maxilla-mandibular complex and concomitant maxillary impaction to correct the skeletal and occlusal deformities. All osteotomies were stabilized using rigid fixation and light intermaxillary elastics.

The treated subjects were matched to untreated subjects who participated in the Montreal Human Growth study. The untreated control group was comprised of French-Canadian children drawn from three school districts representing the different socio-economic strata of the larger population underwent serial lateral cephalograms for comparison purposes [18]. Controls were all hyperdivergent subjects matched to the treated population based on age, sex, and Angle classification. The untreated group consisted of 22 females. The ages of the control and treated groups were not statistically different (Table 1).

### Cephalometric analysis

Using Dolphin Imaging^TM^ 11.0 (Dolphin Imaging and Management Solutions, Chatsworth, CA), three landmarks were identified [19] and digitized, along with the internal structures and external surfaces of the mandible necessary for superimposition purposes (Table 2).

**Table 2:** Cephalometric landmarks [18]

Landmark	Abbreviation	Definition
Sella	S	Center of sella turcica
Nasion	N	Anterior point on the frontonasal suture
Condylion	Co	Point tangential to the most superior aspect of the condyle using a perpendicular to the ramal plane

**Fig. 1:**
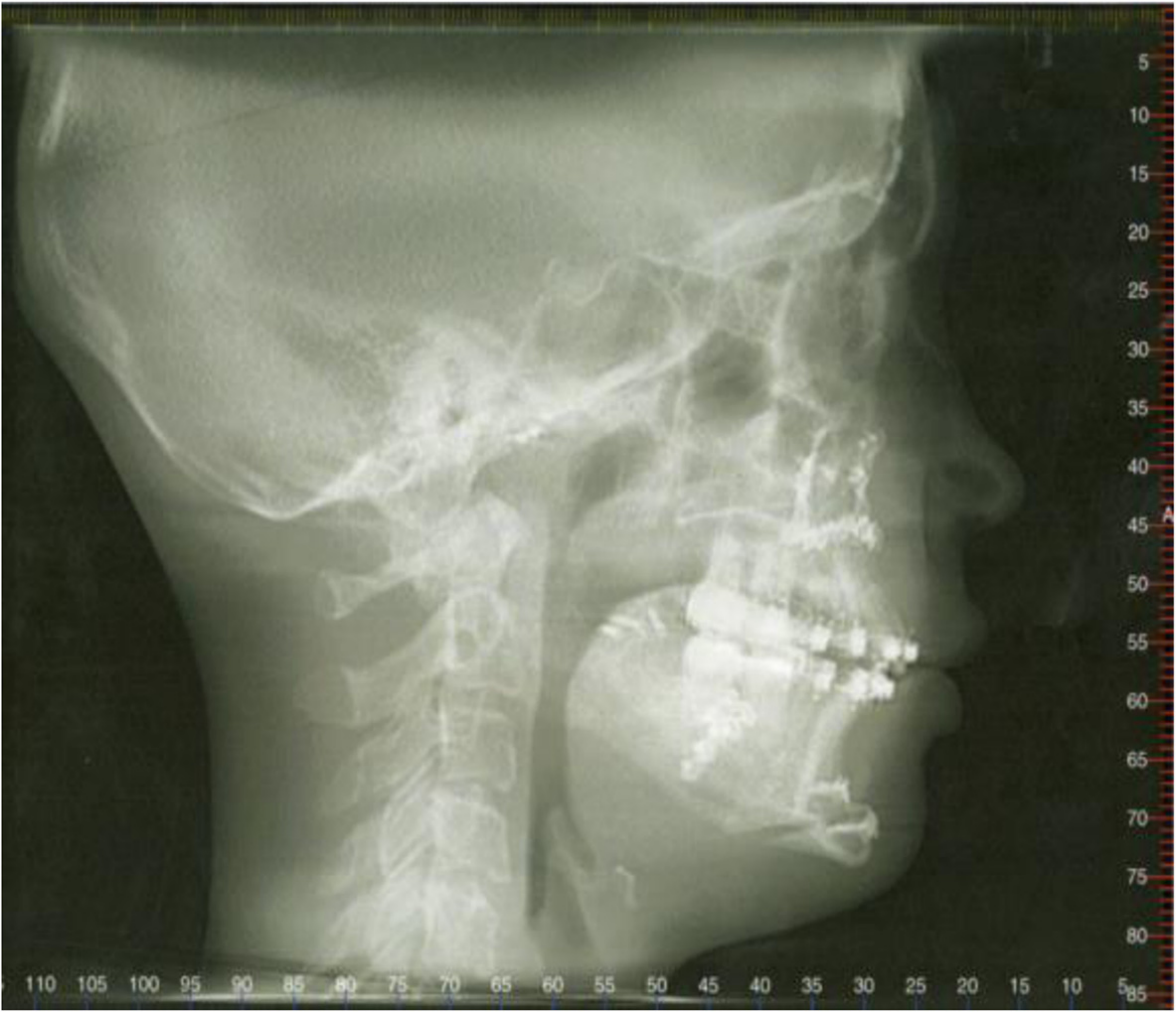
Post-surgical cephalometric radiograph demonstrating the rigid fixation devices in the proximal segment of the mandible used for superimposition

Mandibular superimpositions were performed to evaluate the growth of the cephalometric landmark condylion (Co), defined as the point tangential to the most superior aspect of the condyle using a perpendicular to the ramal plane. The control and pre-surgical tracings were superimposed on the following: (1) inner contour of the cortical plate at the lower border of the symphysis, (2) distinct trabecular structures in the symphysis, and (3) contour of the mandibular canal [20]. The post-surgical superimpositions of the treated cases were performed using the same structures, as well as distinct rigid fixation hardware in the proximal segment of the mandible (Fig. 1).

### Calculations

To quantify the horizontal and vertical changes in Co, reference axes were established on the pre-surgical tracing. The horizontal axis was defined as sella-nasion minus 7°, and a perpendicular to the *x*-axis, registered on sella, defined the *y*-axis. The reference axes made it possible to quantify the horizontal, vertical, and total changes in condylar position. Negative values indicate that Co moved in a posterior or inferior direction. The total change included both the horizontal and vertical (i.e., the hypotenuse) growth changes.

### Measurement reliability

To reduce measurement errors, all cephalograms were digitized and traced by one investigator (TPB). Based on 12 replicates, Cronbach’s alpha (0.941) indicated reliable landmark identification.

### Statistical analysis

Skewness and kurtosis statistics showed that the data were normally distributed. Means and standard deviations (SD) were used to describe the age distributions and condylar growth changes. The growth changes were annualized by dividing each individual’s growth changes by the age changes that occurred. The pre-surgical changes in Co were compared to the post-surgical changes using paired *t* tests. Independent sample *t* tests were performed to determine group differences. All calculations were performed using SPSS Statistics software (version 17.0, SPSS, Chicago, IL) with the significance level set at 0.05.

## Results

### Treated subjects

The 18 treated patients with pre-surgical records showed a decrease in vertical condylar height (0.7 mm/year) and posterior condylar growth (0.5 mm/year). Their total pre-surgical change in Co position was 1.5 mm/year (Table 3). The 15 treated subjects with post-surgical records showed superior (1.4 mm/year) and posterior (0.9 mm/year) growth of Co. The total post-surgical change of Co was 2.0 mm/year. The 11 patients with complete data at all four time points show comparable pre- and post-surgical changes. Comparisons showed that statistically significant differences in vertical (*p* < 0.001), but not horizontal or total, condylar growth occurred between the pre- and post-surgical time periods.

**Table 3:** Pre- (T2-T1) and post-surgical (T4-T3) changes in condylar position of the treatment group, with statistics for all 18 pre-surgical and 15 post-surgical patients shown above and for the 11 patients with complete data at all four time points shown below

	Pre-surgical		Post-surgical	
	Mean (mm/year)	SD	Mean (mm/year)	SD
All available patients				
Total	1.46	1.18	2.00	0.71
*x*-axis^a^	−0.52	0.63	−0.88	1.08
*y*-axis^a^	−0.66	0.61	+1.39	1.12
Patients with complete data				
Total	1.65	1.21	1.96	0.74
*x*-axis^a^	−0.65	0.56	−0.95	0.95
*y*-axis^a^	−0.67	0.71	+1.37	1.27

^a^(+) anteriorly and superiorly; (−) posteriorly and inferiorly

### Untreated subjects

The untreated control subjects showed superior (+1.2 mm/year) and a slight posterior (−0.1 mm/year) growth of Co over the corresponding pre-surgical matched interval (T2-T1) (Table 4). Over the post-surgical time interval (T4-T3), the control subjects continued to show superior (+1.3 mm/year) and anterior (+0.2 mm/year) condylar growth.

### Comparisons

**Fig. 2:**
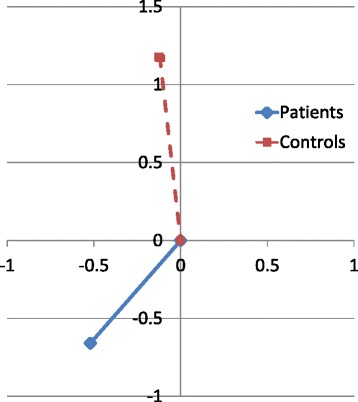
Change in Co (mm) from initial records (T1) plotted at (0,0) to immediate pre-surgical records (T2)

Both the horizontal and vertical changes in Co position showed statistically significant group pre-surgical differences (Table 4). While the control subjects’ condyles grew superiorly, the treated group showed a loss in condylar height. In addition, the treated subjects showed significantly more posterior growth of Co pre-surgically than the control group (Fig. 2). The group difference in the total change during the pre-surgical time period was not statistically significant.

**Table 4:** Pre-and post-surgical growth changes of Co, along with statistical comparisons of the treatment and control groups

	Treatment group		Control group		Prob group differences
	Mean (mm/year)	SD	Mean (mm/year)	SD	
Pre-surgical (T2-T1) changes					
Total	1.46	1.18	1.62	1.46	0.886
*x*-axis^a^	−0.52	0.63	−0.12	0.67	0.041
*y*-axis^a^	−0.66	0.61	+1.18	1.75	0.022
Post-surgical (T4-T3) changes					
Total	2.00	0.71	2.15	1.66	0.473
*x*-axis^a^	−0.88	1.08	+0.16	0.45	0.054
*y*-axis^a^	+1.39	1.12	+1.27	1.23	0.471

^a^(+) anteriorly and superiorly; (−) posteriorly and inferiorly

**Fig. 3:**
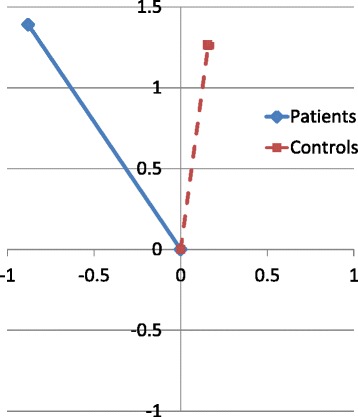
Positional change in Co from immediate post-surgical (T3) plotted at (0,0) to follow-up exam (T4)

The post-surgical changes in condylar position showed no statistically significant group differences. Both groups showed similar amount of superior and total condylar growth (Fig. 3). There were differences in horizontal growth, with the controls exhibiting slight anterior and the treated group showing posterior condylar growth. The average superior growth changes in Co for the treatment and control subjects 1.4 and 1.3 mm/year, respectively (Table 4).

## Discussion

The adolescent patients in the present study diagnosed with AICR and anterior disc displacement showed decreases of condylar height prior to treatment. Loss of vertical height in patients with internal derangement of the TMJ disc has been previously reported. Wolford and Cardenas [10], who followed 12 subjects for 8 months prior to surgery, found 1.1 mm of condylar resorption, 1.8 mm posterior movement of point B, and a 1.5° increase of the mandibular occlusal plane. Cai and coworkers [21], who followed patients with anterior disc displacements for 10.9 months, reported a 0.6-mm decrease in condylar height. Flores-Mir et al. reported reduced vertical growth of the condyle among patients with TMJ disc abnormalities [22]. Animal models with surgically induced anterior disc displacements also develop vertical condylar deficiencies [23].

It has been suggested that internal derangement is the primary etiologic factor leading to deficient mandibular growth [24]. The change in the disc’s orientation relative to the condyle moves its load-bearing zone from the biconcave articular portion to the vascularized posterior band, which has been shown to lead to osteoarthritis and subsequent condylar and mandibular deficiencies [25–27]. Although the specific cause of AICR has not been clearly identified, its strong predilection for pubertal females suggests hormonal mediation. Estrogen receptors have been identified in the TMJs of female primates [28, 29], in human TMJ tissues [30], and in arthritic knee joints [31] Estrogen is known to mediate cartilage and bone metabolism in the female TMJ. An increase in receptors may predispose an exaggerated response in the bilaminar tissues where an increase of synovial cells has been identified in this non-inflammatory pathological condition. Increased TMJ loading from parafunctional activity, trauma, orthodontics, or orthognathic surgery could accelerate the resorption process. Gunson et al. proposed that abnormally low 17 beta-estradiol contributes to condylar lysis [32].

Female hormones mediate biochemical changes within the TMJ bilaminar tissues causing hyperplasia of the synovial tissues that initiate breakdown of the ligamentous structures that normally support and stabilize the articular disc in position. This allows the disc to become anteriorly displaced. The hyperplastic synovial tissue then surrounds the head of the condyle, with destructive substrates penetrating through the fibrocartilage, creating an internal condylar resorptive phenomena by breaking down the subcortical and medullary bone. The condyle decreases in size without apparent destruction of the fibrocartilage on the condylar head and fossa. This is unlike all other arthritides, where the fibrocartilage is destroyed by the inflammatory disease processes. AICR is a non-inflammatory process that can progress for a while and then go into remission or proceed until the entire condylar head has resorbed. In cases where it goes into remission, excessive joint loading (i.e., parafunctional habits, trauma, orthodontics, orthognathic surgery) can reinitiate the resorption process at a later date.

The treated subjects displayed greater than expected posterior condylar growth. Although unexpected, this result coincides with the work done by Björk and Skieller [20], who showed that backward rotation of the mandible is associated with a more posterior condylar growth. All of the treated subjects had extremely large mandibular plane angles, indicative of backward rotation. Ikeda and Kawamura [33], who used MRIs to assess condylar positional changes in joints with disc displacement, showed that the condyles were positioned more posteriorly in the fossa than in normal joints. Posterior condylar growth, along with loss of condylar height, could contribute to the morphologic features typically described by those who study ICR [10, 28–35].

After undergoing both disc repositioning and concomitant orthognathic surgery, the patients showed a dramatic redirection of condylar growth. Post-surgically, the patients showed superior growth of the condyle similar to that of the control subjects. Many factors could influence this change in condylar growth direction following surgery. Unlike other TMD procedures, such as splint therapy, arthrocentesis, or arthroscopy, the Mitek anchor surgery technique removes the hyperplastic synovial tissue that surrounds the condyle and eliminates the TMJ pathosis [12]. Disc repositioning with Mitek anchors significantly improves patients’ reported TMJ/facial pain and headaches, as well as jaw function [10, 12, 36].

The cessation of the AICR along with reestablishing the proper functional articulation may produce an environment in which the condyle resumes its normal growth. Adaptive compensations in mandibular growth following orthognathic surgery or orthodontic therapy could explain the alteration in the direction of condylar growth. Superior maxillary repositioning by LeFort I osteotomy alters post-surgical mandibular remodeling and condylar growth [37]. These alterations appeared to be adaptive compensations for the surgical repositioning and counter-clockwise rotation of the mandible. Since all of the patients in the current study underwent both mandibular BSSO advancement and maxillary LeFort I procedures, with decreases of the occlusal and mandibular plane angles, adaptive compensations of the condyle may contribute to the alteration in the direction of growth.

Condylar growth in both the vertical and horizontal planes was normalized following surgery. Based on published incremental condylar growth charts [38], the treated patients fall in the 80th percentile for condylar growth of 15.5-year-olds. This is an important result when evaluating the ramifications of operating on the joints of growing individuals. Wolford and others have reported that Mitek anchors are stable and provide good surgical outcomes [12, 13, 39, 40]. Patients with AICR are typically girls in their pubertal growth phase [10]. Given the timing of the onset of this disease process, some patients may be seeking or be active in orthodontic treatment when the detrimental effects begin.

Since the Mitek anchor osseo-integrates with the condyle, it becomes a fixed bone implant in the condylar head [39]. The condylar growth occurs above the level of the Mitek anchor. Therefore, the age of surgical intervention must be carefully considered in growing patients. If Mitek anchor surgery is done too early, the condyle may grow significantly upward away from the anchor and could result in posterior disc displacement by the time normal growth ends. For most surgeons, it would be better to perform the orthognathic surgery at 15 for females and 17 for males, after the majority of facial growth is complete. The disc repositioning with the Mitek anchor should ideally not be performed earlier than 14 in females and 16 in males. The TMJ surgery can be done as a separate procedure but should be performed prior to orthognathic surgery when done concomitantly.

Due to the study’s limitations, the results should be considered as preliminary. The size of the treated sample, especially the subsample of patients with complete data, was small. This reduces the power of the statistical tests. Fortunately, the surgical effect was substantial and the differences were significant. The historical controls that were used introduce possible selection bias. This emphasizes the importance of having pre-surgical changes available against which to evaluate the post-surgical changes. Due to these limitations, further, better controlled studies should be performed.

## Conclusions

Based on a treated sample of 22 female adolescents aged 9–15 pre-surgically and followed for at least 1 year post-surgically, and an untreated sample of 22 age-matched female adolescents, the following conclusions can be drawn:

1. Patients diagnosed with AICR and anterior disc displacement show abnormal condylar growth prior to surgical intervention.
2. Following successful repositioning of the disc by means of the Mitek mini-anchor system, patients show a normalized growth pattern of the condyle.
